# The Types of Bullying Behaviors and Its Association with General Life Satisfaction and Self-Rated Health among Iranian Pupils

**Published:** 2019-01-09

**Authors:** Maysam Rezapour, Narges Khanjan, Hamid Soori

**Affiliations:** ^1^ Neurology Research Center, Department of Epidemiology and Biostatistics, School of Public Health, Kerman University of Medical Sciences, Kerman, Iran; ^2^ Safety Promotion and Injury Prevention Research Center, Shahid Beheshti University of Medical Sciences, Tehran, Iran

**Keywords:** Bullying, Quality of life, Self-assessment, Health status, Pupils

## Abstract

**Background:** Bullying in school-age children is a public health concern that less discusses in Middle East countries like Iran. The goal of this study was to determine and compare whether exposure to various forms of bullying behaviors contributes to disparities in life satisfaction and self-rated health among Iranian pupils.

**Study design:** A cross-sectional study.

**Methods:** Data were obtained from a cross-sectional study on 834, 8th and 9th-grade students conducted in northern Iran in 2014. Bullying was measured by the Iranian-version of the Olweus Bullying Questionnaire. Life satisfaction and self-rated health were assessed by a single item of the Global School Health Survey (GSHS) in Persian. Descriptive statistics and two-level logistic regression analysis were used for data analysis.

**Results:** Positive self-rated health and life satisfaction were significantly higher in boys than girls (*P*<0.002). Self-related health and life satisfaction were similar in the 8th and 9th-grade students. After controlling for gender and grade, students involved in only-victim of verbal bullying (OR=0.48, 95% CI: 0.34, 0.67), and bully-victim of relational bullying (OR=0.29, 95% CI: 0.12, 0.67) reported lower selfrated health compared to non-involved students.

**Conclusion:** Life satisfaction and self-rated health were differently related to types of bullying behaviors. Our findings highlighted the necessity to detect victims and bully-victims and develop prevention programs to stop bullying and its negative consequences in Iranian schools.

## Introduction

Bullying in school-age children is a public health concern. According to the definition of CDC^1^, “bullying is any unwanted aggressive behavior(s) by another youth or group of youths who are not siblings or current dating partners, which involves an observed or perceived power imbalance and is repeated multiple times or is highly likely to be repeated”. Students involved in bullying behaviors are often divided into 3 categories; only-victims, only-bullies, and bully-victims and each of them include various forms, such as physical (e.g., hitting, pushing, and kicking), verbal (e.g., name-calling and teasing in a hurtful way), relational or social (e.g. social exclusion and spreading rumors) and cyber (e.g. mobile phones, internet, email, online social networking or creating nasty websites) ^1-4^.

The prevalence of bullying behaviors in western countries shows varying estimates; from 5% in Sweden to 44% in New Zealand for bullies, and 7% in Switzerland to 43% in Italy for victims ^5^. In Iran, 5.4%, 22.1%, and 11.0% of all students were involved in only-bully, only-victim, and bully-victims, respectively; and the verbal and cyber forms of the noted categories were the most and least common, respectively ^6^. Bullying is associated with a number of behavioral, emotional and physical adjustment problems^7-13^. Only-victims, only-bullies, and bully-victims reported higher health problems than those not involved in bullying behaviors ^14^. In this meta-analysis, only-victims and bully-victims reported the largest effect sizes, whereas only-bullies reported lower health problems than the former two groups ^14^. Although, various forms of bullying are related; but their occurrence circumstances, nature, and even negative health consequences may diffe ^15^.

Self-rated health is a person’s subjective measurement of his/her own overall well-being. It is one of the most widely used measures in public health and is associated with a broad range of health indicators including medical, psychological, social and health behaviors ^16^. Long-term sickness can reduce self-rated health ^17^. In a trend analysis on data coming from 32 countries (mostly European) from 2002 to 2010 about Health Behavior in School-Aged Children, girls were compared to their male peers constantly rated their health as poorer, in all countries and their rating of health also decreased with increasing age^18^. Experiencing bullying behaviours is associated with poor health expressed in the form of emotional and physical adjustment problems ^7-11^.

Life satisfaction is one of the components that contribute to the development of psychological well-being. Life satisfaction describes participants’ personal evaluation of their contentment and views about their current life circumstances^19^. Life satisfaction is related to a number of factors such as personal characteristics, familial and peer relations. Involvements in bullying behaviours are associated with lower life satisfaction ^20-22^

Most previous studies focused on the relation between victimization and perpetration of bullying with life satisfaction^23,24^ and self-rated general health^25,26^. There is little evidence on the relation of various forms of bullying in the only-victim, only-bully, and bully-victim categories with life satisfaction and self-rated general health. Considering no previous study on this topic in Iran, the main objective of this study was examining pupil’s experiences about different types of bullying and how these forms of bullying are associated with life satisfaction and self-rated health.

## Methods

### Data Sample

Overall, 834 pupils (boy and girls) from the 8^th^ and 9^th^ grade were randomly selected from 16 public schools of Mazandaran Province, northern Iran, by stratified clustered sampling and based on data from the 2013 project of school bullying in Mazandaran, Iran.

### Measures

Students’ experiences about bullying behaviors (victimization or perpetration) during the past three months was measured by the Persian- Olweus Bullying Questionnaire (P-OBQ) that is a modified version of the Olweus Bullying Questionnaire (OBQ) and has been validated among Iranian pupils ^27^. In this questionnaire, victimization was assessed by 10 items (Cronbach’s alpha=0.80), which asks students to report how often they have been bullied at school during the past three months by one or more of the noted ways. Perpetration of bullying was assessed by 10 items (Cronbach’s Alpha=0.81), which asks students to indicate how often they have taken part in bullying another student(s) at school during the past three months by one or more of the noted ways. Items were scored on a 5-point scale (1=never, 2=once or twice, 3=2 or 3 times a month, 4=once a week, 5=several times a week). Four-factor structure (verbal, relational, physical and cyber) of victimization and perpetration of bullying has sufficient validity ^27^. The cutoff point of ‘‘2 or 3 times a month’’ was recommended as the most suitable cutoff point for dividing the population into involved and uninvolved in bullying^28^. According to this cutoff point pupils were categorized into four groups as not-involved, only victims, only bullies and bully-victims; and in verbal, relational, physical, and cyber forms of bullying.

Self-rated health and Life satisfaction were measured by the Global School Health Survey (GSHS) in Persian and the validity of questions has been assessed previously^29,30^.

Self-rated health was measured using a single-item Likert scale (How do you rate your health in general?) and collected at four levels (“excellent”, “good”, “fair” or “poor”). This variable was subsequently dichotomized for analysis, by combining the first two (positive self-rated health) and the last two categories (negative self-rated health). Life satisfaction was measured using the 10 steps of the Cantril’s ladder (0 as the worst possible life situation to 10 as the best possible). Pupils were asked to show where they would place their lives in regard to “life satisfaction” at present. A Cantril’s score of 6 and over was considered as fair.

### Procedure

Permission to conduct this study was obtained from the Educational Authority of Mazandaran Province and informed consent was obtained from the parents and teachers of the selected schools.

Data were collected in the classrooms by the cooperation of school teachers using anonymous self-reported questionnaires to ensure confidentiality. After distributing the questionnaires, the researcher described a definition of bullying and the purpose and importance of the study for participants. Students were asked to complete the questionnaires on a voluntary and honest basis. The data were gathered in Feb and Mar 2014.

### Data Analysis

The associations of self-rated health and life satisfaction with gender, grade levels, and the different categories (only bully, only victim, bully-victim, and non-involved) of various bullying forms (verbal, relational, physical, and cyber) were assessed using Pearson's chi-square test. For each outcome, we used two-level (random intercept) logistic regression analyses with robust variance for each of the forms of bullying adjusted for gender, and grade in 4 models, separately (Model 1: verbal bullying, Model 2: relational bullying, Model 3: physical bullying, and Model 4: cyber bullying) . Each of bullying forms was divided into four categories: only bully, only victim, bully-victim, and not involved. Not-involved was set as the reference group. Analyses were conducted using Stata 12.

## Results

Table 1 shows the frequency and percentage of self-related health and life satisfaction across gender and grade groups. Positive self-rated health and poor life satisfaction were significantly higher in boys than girls. Self-related health and life satisfaction were similar in the 8^th^ and 9^th^-grade students. There was a significant association between verbal and relational bullying and self-rated health.

**Table1:** The frequency (%) of Self-related health, and Life satisfaction across gender and grade, and different types of bullying

**Variables**	**Total,** **n (%)**	**Self-rated health,** **n (%)**		**Life satisfaction,** **n (%)**	
		**Negative**	**Positive**	**Poor**	**Fair**
Gender					
Girl	435 (52.2)	122 (28.3)	309 (71.7)	120 (27.8)	311 (72.2)
Boy	399 (47.8)	70 (17.9)	321 (82.1)	74 (18.7)	321 (81.3)
*P* value		0.001		0.002	
Grade					
8th	429 (51.4)	105 (24.8)	318 (75.2)	92 (21.7)	332 (78.3)
9th	405 (48.6)	87 (21.8)	312 (78.2)	102 (25.4)	300 (74.6)
*P* value		0.307		0.213	
Verbal bullying					
Not-involved	511 (62.8)	103 (20.4)	403 (79.6)	109 (21.4)	401 (78.6)
Only-bully	44 (5.4)	10 (23.8)	32 (76.2)	13 (30.9)	29 (69.1)
Only-victim	184 (22.6)	57 (31.5)	124 (68.5)	49 (27.2)	131 (72.8)
Bully-victim	75 (9.2)	19 (25.7)	55 (74.3)	17 (22.7)	58 (77.3)
*P* value		0.024		0.261	
Relational bullying					
Not-involved	643 (78.5)	142 (22.4)	492 (77.6)	139 (21.7)	500 (78.3)
Only-bully	24 (2.9)	5 (20.8)	19 (79.2)	4 (16.7)	20 (83.3)
Only-victim	122 (14.9)	28 (23.3)	92 (76.7)	36 (30.5)	82 (69.5)
Bully-victim	30 (3.7)	14 (46.7)	16 (53.3)	9 (30.0)	21 (70.0)
*P* value		0.023		0.130	
Physical bullying					
Not-involved	640 (77.9)	143 (22.7)	487 (77.3)	14 4(22.6)	492 (77.4)
Only-bully	35 (4.3)	9 (25.7)	26 (74.3)	8 (23.5)	26 (76.5)
Only-victim	108 (13.1)	27 (25.2)	80 (74.8)	27 (25.5)	79 (74.5)
Bully-victim	39 (4.7)	10 (26.3)	28 (73.7)	9 (23.7)	29 (76.3)
*P* value		0.883		0.936	
Cyber bullying					
Not-involved	792 (95.5)	179 (22.9)	602 (77.1)	182 (23.2)	603 (76.8)
Only-bully	17 (2.1)	5 (31.3)	11 (68.7)	4 (23.5)	13 (76.5)
Only-victim	15 (1.8)	3 (20.0)	12 (80.0)	4 (26.7)	11 (73.3)
Bully-victim	5 (0.6)	3 (60.0)	2 (40.0)	1 (25.0)	3 (75.0)
*P* value		0.213		0.991	

A multilevel logistic regression model without predictors was run to examine whether self-rated health and life satisfaction varied between schools, by variance component models; and intraclass correlation coefficients (ICCs) (ICC = σ2class level / (σ2class level+3.29)) were calculated. The ICCs of self-rated health (3.6%) and life satisfaction (4.1%) showed that most of the variation in these variables were found at an individual level. Then, gender and grade were added as confounder variables and each bullying form was set as main exposure variable. The result of two-level logistic regression analyses about the association between various bullying forms and self-rated health and also life satisfaction, after controlling for gender and grade are presented in Table 2.

**Table 2:** Odds ratios (OR) of association between the types of bullying and self-rated health and life satisfaction

**Variables**	**Odds ratio (95%CI)**	
	**Self-rated health**	**Life satisfaction**
Model 1		
Gender (Ref: girls)	2.10 (1.39, 3.16)	1.86 (1.20, 2.90)
Grade (Ref: 8^th^ )	1.23 (0.85, 1.78)	0.81 (0.55, 1.18)
Verbal bullying (Ref: not-involved)		
Only-bully	0.71 (0.33, 1.49)	0.53 (0.25, 1.11)
Only-victim	0.48 (0.34, 0.67)	0.62 (0.41, 0.94)
Bully-victim	0.56 (0.26, 1.13)	0.71 (0.41, 1.19)
Variance of random intercept (ICC %)	0.002 (0.06%)	0.055 (1.40%)
Model 2		
Gender(ref: girls)	1.89 (1.30-2.74)	1.70 (1.13, 2.55)
Grade(ref: 8^th^ )	1.24 (0.83-1.84)	0.82 (0.56, 1.18)
Relational bullying (Ref: not-involved)	
Only-bully	0.92 (0.31-2.83)	1.15 (0.27, 4.96)
Only-victim	0.92 (0.61, 1.41)	0.59 (0.35, 1.02)
Bully-victim	0.29 (0.12, 0.67)	0.58 (0.25, 1.31)
Variance of random intercept (ICC %)	0.004 (0.12%)	0.035 (1.11%)
Model 3		
Gender (Ref: girls)	1.85 (1.22, 2.81)	1.73 (1.11, 2.72)
Grade (Ref: 8^th^ )	1.22 (0.82, 1.81)	0.83 (0.56, 1.23)
physical bullying (Ref: not-involved)		
Only-bully	0.66 (0.32, 1.34)	0.76 (0.36, 1.58)
Only-victim	0.74 (0.48, 1.14)	0.75 (0.44, 1.30)
Bully-victim	0.71 (0.34, 1.47)	0.72 (0.37, 1.42)
Variance of random intercept (ICC %)	0.034 (1.01%)	0.059 (1.71%)
Model 4		
Gender (Ref: girls)	1.88 (1.31, 2.68)	1.68 (1.12, 2.52)
Grade (Ref: 8^th^ )	1.21 (0.83, 1.75)	0.82 (0.57, 1.19)
Cyber bullying (Ref: not-involved)		
Only-bully	0.51 (0.21, 1.22)	0.74 (0.25, 2.20)
Only-victim	1.13 (0.30, 4.22)	0.72 (0.22, 2.37)
Bully-victim	0.17 (0.03, 1.12)	0.77 (0.07, 8.21)
Variance of random intercept (ICC %)	0.001 (0.01%)	0.026 (0.78%)

Model 1 shows self-rated health and life satisfaction are related to victimization (only-victim) in the verbal form OR=0.48 (95% CI: 0.34, 0.67) for self-rated health and OR=0.62 (95% CI: 0.41, 0.94) for life ratification.

Model 2 shows involvement in bully-victim of relational bullying is related to lower self-rated health OR=0.29 (95% CI: 0.12, 0.67), while there is no association between relational bullying and life satisfaction. Model 3 and 4 show no association of physical bullying and cyberbullying with self-rated health and life satisfaction either.

Grade was not associated with self-rated health and life satisfaction in any model, while boys reported higher self-rated health and life satisfaction than girls in all models (ranged from OR=1.85 (95% CI: 1.22, 2.81) in model 3 to OR=2.10 (95% CI: 1.39, 3.16) in model 1for self-rated health and OR=1.68 (95% CI: 1.12, 2.52) in model 4 to OR=1.86 (95% CI: 1.20, 2.90) in model 1for life satisfaction.

The ICCs of self-rated health and life satisfaction in each form of bullying showed that the differences between schools were explained to some extent by individual-level variables, since the proportion of variance (ICCs) in self-rated health and life satisfaction explained by differences between schools decreased in comparison to the model without any variables (Table 2).

## Discussion

In this cross-sectional study, we compared the associations between various forms of bullying and life satisfaction and self-rated general health, among Iranian pupils. Consistent with a previous national survey of school student high-risk behaviors’’(2009–2010) conducted in Iran ^31^, the current study showed fair life satisfaction was seen more in boys, while poor life satisfaction was more prevalent among girl. But negative self-rated health was seen more in girls, while positive self-rated health was more in boys.

The findings of this study showed that involvement in only-bully of all forms is not significantly associated with reduced self-rated health and life satisfaction. This may be because of the characteristics of only-bullies such as high popularity, self-esteem, and social skills ^32^. While involvement as only-victim in the verbal form was associated with lower reports of self-rated health and life satisfaction, but involvement as only-victim in the relational, physical and cyber forms were not related to significant decreases in self-rated health and life satisfaction. These results may be because of the characteristics of only-victims in the verbal form compared with only-victims of other forms. Only-victims in the verbal form was showed experience higher psychosomatic problems than only-victims of the relational and cyber forms^33^.

Our results showed involvement as bully-victim in the relational form reduces self-rated health, while involvement as bully-victim in other forms was not associated with self-rated health. In addition, involvement as bully-victim in various forms (verbal, relational, physical, and cyber) was not associated with life satisfaction either.

Consistent with our findings, in Pennsylvania State, students who were victims of bullying reported less life satisfaction than students not involved ^23^. Moreover, in Swedish pupils, in both boys and girls, there were strong associations between poor general health and bullying behaviors ^26^. In addition, students who bully and/or are bullied experience less life satisfaction compared to children who are neither victims nor perpetrators of bullying^24^.

The results of this study showed no association between physical bullying and life satisfaction and self-rated health. However, physical harassment at school was positively associated with poor health (OR=0.43, *P*-value<0.001), while harassment outside school was not (OR=1.09, *P*-value>0.05)^25^.

A study on 855, grades 7 and 8 students in southeastern USA ^34^, showed that after controlling for demographic (gender and grade) variables, electronic bullies had significantly less global life satisfaction (OR=0.86, *P*-value<0.001); but the relation between electronic victimization and global life satisfaction was non-significant. Whereas in the current study, involvement in only-victim and only-bully of cyberbullying showed no significant association with life satisfaction. Inconsistent with our results, a study on 497, 9 to 12-grade students in public and private Chilean schools showed the negative impacts of traditional (verbal, relational, and physical) and cyber victimization on life satisfaction ^22^.

One factor explaining the inconsistency in our results with studies from other countries is related to the cultural context of Iranian schools, which needs further research.

Bullying behaviors are usually recognized as complicated social phenomena resulting from individual, peer group, and broader social interactions ^35^. In this study, the ICCs of self-reported health and life satisfaction in all models were relatively small; because according to the multilevel approach, almost all of the variability in self-reported health and life satisfaction between schools was explained by differences between individuals.

The present study had some limitations. First of all, data was measured by self-reported questionnaires. Second, the finding of the current study was based on overall life satisfaction and self-rated health, which may mask the effects of their specific domains ^34^. Thus, further research is needed to evaluate domain-based life satisfaction and self-rated health measures in Iranian schools. Third, the cross-sectional nature of study does not allow us to make any causal inference about the association between bullying and health problems. Finally, this study was conducted in only one province of Iran and in public schools, which limits its generalizability.

Implications for practice must be taken cautiously. This study presents valuable information for school counselors, health care professionals, school staff, and parents. Our findings highlight the necessity to detect victims and bully-victims by a surveillance system^1^ and develop prevention programs to stop bullying and its negative consequences in Iranian schools.

## Conclusion

This study provides further evidence about the impact of various bullying forms on pupils’ health perception. Life satisfaction and self-rated health were differently related to types of bullying behaviors. In other words, except victimization in the verbal bullying form and bully-victim in relational bullying; other categories of various forms of bullying were not associated with global life satisfaction and self-rated health. In addition, this study did not show an association between experiencing physical, and cyber forms of bullying and self-rated health or poor life satisfaction.

## Acknowledgements

We thank all the students and school staff for participating in this program. We acknowledge the Security Office of the Education Authority of Mazandaran Province, Iran for their support.

## Conflict of interest statement

The authors declare that there is no conflict of interests.

## Funding

The study was supported by the Safety Promotion and Injury Prevention Research Center of Shahid Beheshti University of Medical Science.

## Highlights

- Bullying is an important problem for school-age children.
- Various forms (verbal, relational, physical and cyber) of bullying behaviors (only-victim, only-bully, and bully-victim) have different impacts on life satisfaction and self-rated health among adolescences.
- The developing prevention programs to stop bullying and its negative consequences are essential in Iranian schools.
- The types of bullying behaviors and its association with general life satisfaction and self-rated health among Iranian pupils
